# IP_3_R1 deficiency in the cerebellum/brainstem causes basal ganglia-independent dystonia by triggering tonic Purkinje cell firings in mice

**DOI:** 10.3389/fncir.2013.00156

**Published:** 2013-10-04

**Authors:** Chihiro Hisatsune, Hiroyuki Miyamoto, Moritoshi Hirono, Naohide Yamaguchi, Takeyuki Sugawara, Naoko Ogawa, Etsuko Ebisui, Toshio Ohshima, Masahisa Yamada, Takao K. Hensch, Mitsuharu Hattori, Katsuhiko Mikoshiba

**Affiliations:** ^1^Laboratory for Developmental Neurobiology, RIKEN Brain Science Institute, Wako, Japan; ^2^Neuronal Circuit Development, RIKEN Brain Science Institute, Wako, Japan; ^3^Precursory Research for Embryonic Science and Technology (PRESTO), Japan Science and Technology Agency (JST), Kawaguchi, Japan; ^4^Yamada Research Unit, RIKEN Brain Science Institute, Wako, Japan; ^5^Department of Life Science and Medical Bio-Science, Waseda University, Tokyo, Japan; ^6^Department of Biomedical Science, Graduate School of Pharmaceutical Sciences, Nagoya City University, Nagoya, Japan; ^7^Calcium Oscillation Project, ICORP-SORST, Japan Science and Technology Agency (JST), Kawaguchi, Japan

**Keywords:** dystonia, Purkinje cells, inferior olive, cerebellum, basal ganglia, complex spikes, inositol 1,4,5-trisphosphate, SCA15

## Abstract

The type 1 inositol 1,4,5- trisphosphate receptor (IP_3_R1) is a Ca^2+^ channel on the endoplasmic reticulum and is a predominant isoform in the brain among the three types of IP_3_Rs. Mice lacking IP_3_R1 show seizure-like behavior; however the cellular and neural circuit mechanism by which IP_3_R1 deletion causes the abnormal movements is unknown. Here, we found that the conditional knockout mice lacking IP_3_R1 specifically in the cerebellum and brainstem experience dystonia and show that cerebellar Purkinje cell (PC) firing patterns were coupled to specific dystonic movements. Recordings in freely behaving mice revealed epochs of low and high frequency PC complex spikes linked to body extension and rigidity, respectively. Remarkably, dystonic symptoms were independent of the basal ganglia, and could be rescued by inactivation of the cerebellum, inferior olive or in the absence of PCs. These findings implicate IP_3_R1-dependent PC firing patterns in cerebellum in motor coordination and the expression of dystonia through the olivo-cerebellar pathway.

## Introduction

The inositol 1,4,5- trisphosphate receptors (IP_3_Rs) are intracellular Ca^2+^ channels localized at the endoplasmic reticulum and regulate the spatio-temporal change of intracellular Ca^2+^ concentration, which are important for diverse physiological phenomena including gene expression, development, growth, neural plasticity, and secretion (Berridge et al., 2000). There are three subtypes of IP_3_R in mammals and each IP_3_R isoform exhibits a distinct expression pattern *in vivo*. Among the three subtypes, the type 1 IP_3_ receptor (IP_3_R1) is a brain dominant subtype (Foskett et al., 2007; Mikoshiba, 2007). We have previously showed that mice lacking the IP_3_R1 receptor (*Itpr1*^−/−^) exhibit ataxia and seizure-like posture with multiple abnormal movements such as repetitive rigid posture, opisthotonus, tonic contractions of the neck and trunk, and premature death around the third week after birth (Matsumoto et al., 1996). However, since IP_3_R1 is expressed in a wide range of brain regions including cerebellum, cerebral cortex, hippocampus, and striatum, the particular neural activities and circuits causing these involuntary movements in the *Itpr1*^−/−^ mice remain unknown.

Dystonia is a neurological disorder in which sustained muscle contractions induce twisting and repetitive movements or abnormal posturing. Simultaneous abnormal contractions of agonistic and antagonistic muscles (co-contractions) are one of the most distinct features of dystonic movements. Because of various phenotypic and genotypic subtypes in dystonia, its pathogenic mechanisms remain elusive. Traditionally, dystonia has been thought to be a basal ganglia (BG) disorder (Marsden and Quinn, 1990; Lenz et al., 1998; Vitek et al., 1999; Zhuang et al., 2004; Chiken et al., 2008; Nambu et al., 2011). In contrast, recent accumulating evidence has further suggested abnormalities of the cerebellum and brainstem in some dystonic patients (Ceballos-Baumann et al., 1995; Eidelberg et al., 1998; Mazziotta et al., 1998; Odergren et al., 1998; Hutchinson et al., 2000). Several animal models of dystonia also exhibit cerebellar abnormalities (Ledoux and Lorden, 2002; Pizoli et al., 2002; Raike et al., 2005; Walter et al., 2006; Chen et al., 2009; Calderon et al., 2011; Ledoux, 2011; Filip et al., 2013) and aberrant cerebellar activities in dystonic model animals were reported (Ledoux and Lorden, 2002; Walter et al., 2006; Chen et al., 2009), however, little is known about firing patterns of Purkinje cell (PC) activity associated with particular dystonic movements of freely moving mice. At the neural circuit level, it was suggested that cerebellar outputs alter BG activity thereby leading to dystonic movements (Neychev et al., 2008; Calderon et al., 2011).

**Figure 1:**
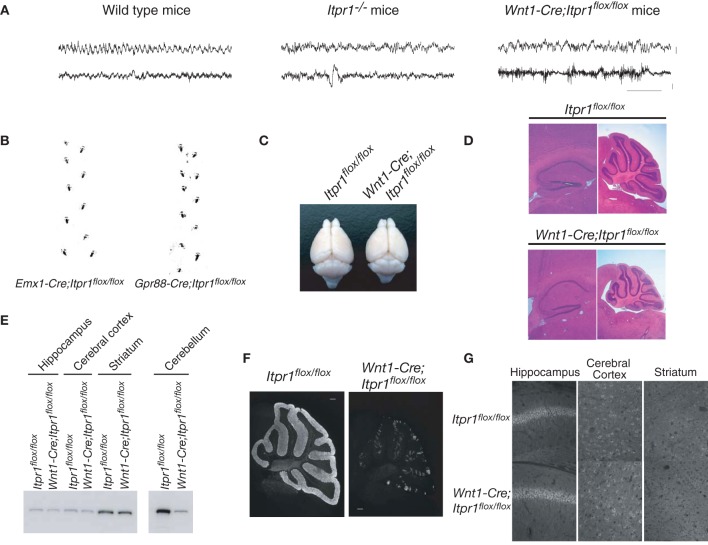
**Cerebellum/brainstem specific IP3R1 deficient mice exhibit dystonia**. **(A)** Electroencephalogram of total *Itpr1*^−/−^ and *Wnt1-Cre;Itpr1^flox/flox^* mice during seizure-like posture. Upper panel showed EEG (bar: 0.1 mV). Lower panel showed EMG (bar: 0.2 mV). Horizontal bar indicates 1.0 s. **(B)** Footprints of *Emx1-Cre;Itpr1^flox/flox^* and *Gpr88-Cre; Itpr1^flox/flox^* mice at 8 weeks. **(C)** Gross appearance of the brain from *Wnt1-Cre;Itpr1^flox/flox^* mice at 8 weeks. **(D)** Hematoxylin and Eosin (HE) staining of the hippocampus and the cerebellum in *Wnt1-Cre;Itpr1^flox/flox^* mice. Note overall size decrease of cerebellum in *Wnt1-Cre;Itpr1^flox/flox^* mice, whereas size of the hippocampus was comparable to *Itpr1^flox/flox^* mice. **(E)** Expression level of IP_3_R1 in various parts of the brain at 8 weeks. **(F)** Immunohistochemical analysis of IP_3_R1 expression in the cerebellum from *Wnt1-Cre;Itpr1^flox/flox^* mice at 8 weeks. **(G)** Immunohistochemistry of the IP_3_R1 expression in the hippocampus, the cerebral cortex, and the striatum from *Itpr1^flox/flox^* (upper panels) and *Wnt1-Cre;Itpr1^flox/flox^* mice (lower panels) at 8 weeks. At least three mice were used for each analysis, and the representative data were shown.

In this study, we showed that genetic deletion of IP_3_R1 within the cerebellum and brainstem is sufficient to cause dystonia in mice. Although in the previous report we described epileptic-like seizures in *Itpr1*^−/−^ mice, in the current study we concluded that the behavior of *Itpr1*^−/−^ mice is better described as dystonia with severe ataxia because there was no abnormal electroencephalogram activity during the seizure-like posture (Figure 1A). In addition, we revealed distinct patterns of PC firing that were tightly coupled to the dystonic movements in freely moving mutant mice. We also showed that pharmacological inactivation of the cerebellum or inferior olive (IO), but not BG, and deletion of PCs ameliorate the dyskinesia. Thus, our mutant mice provide a therapeutic dystonia model solely dependent upon abnormal olivocerebellar pathways and provide a coherent mechanism for a specific type of dystonia.

## Materials and methods

### Mice

For generation of conditional *Itpr1* knockout mice, the floxed *Itpr1* mice (Sugawara et al., 2013) were crossed with *Wnt1 promoter-Cre* (Danielian et al., 1998), *Emx1-Cre* (Iwasato et al., 2000), and *Gpr88-Cre* transgenic (Tg) mice (Hisatsune et al., 2013). Genotyping of *Lurcher* mice was performed as previously (Nishiyama et al., 2010). Body size/weight of *Emx1-Cre;Itpr1^flox/flox^* and *Gpr88-Cre*;*Itpr1^flox/flox^* was indistinguishable from that of control mice. The conditional mice lacking IP_3_R1 in the cerebellum/brainstem (*Wnt1-Cre;Itpr1^flox/flox^* mice) were severely dystonic and could not take in enough food to survive, so they were hand-fed a nutritionally complete soft diet, DietGel76A (ClearH_2_O), for their entire lives after weaning. All animals were ethically treated according to the guideline of Animal Experiments Committee of RIKEN Brain Science Institute.

### Histology and *in-situ* hybridization

The mice brains (18- to 20-day-old *Itpr1*^−/−^ and littermate *Itpr1*^+/+^ mice; 8-week-old *Wnt1-Cre;Itpr1^flox/flox^* and *Itpr1^flox/flox^* mice; and 23-day-old mice for *Lurcher* experiments) were transcardially perfused with 4% PFA in PBS. The fixed brains were immersed in 30% sucrose in PBS for O/N at 4°C. The brains were quickly frozen in Tissue-Tek compound (SAKURA, Japan), and cryostat sections (12 μm in thick) were made.

For immunohistochemistry, the sections were permeablized with 0.25% Triton/PBS for 5 min and immersed with boiled acetate buffer (10 mM, pH = 6.0) for 10 min. After blocked, the sections were probed with the indicated primary antibodies [anti-IP_3_R1 antibodies (18A10, 5.0 μg/ml), rabbit anti-Tyrosine hydroxylase (TH) antibodies (1.0 μg/ml), rabbit anti-Homer 3 S120 antibody, and guinea pig anti-Homer 3 antibodies] for ON at 4°C. After washed with PBS, the sections were probed with Alexa 594-conjugated goat anti-rabbit IgG, Alexa 488-conjugated anti-guinea pig IgG, and Alexa 488-conjugated goat anti-rat IgG (Invitrogen) for 1 h at RT. The coverslips were mounted with Vectashield (Vector Laboratories) and observed under fluorescence microscopy E600 (Nikon).

For *in-situ* hybridization, frozen (12 μm thick) or paraffin-embedded (5 μm thick) sections of 18- to 20-day-old *Itpr1*^−/−^ and *Itpr1*^+/+^ mice or 8-week-old and 19-day-old *Wnt1-Cre;Itpr1^flox/flox^* and *Itpr1^flox/flox^* mice were treated with proteinase K (1 μg/ml, Wako) for 10 min at RT. The sections were blocked and probed with sense and antisense *cfos* probes for ON at 68°C. The *cfos* fragment was amplified with primers, sense primer: 5′-CCGAATTCATGATGTTCTCGGGTTTCAACG-3′, anti-sense primer: 5′-CCAAGCTTTCACAGGGCCAGCAGCGTGG-3′. The underlines indicate *Eco*RI and *Hind*III sites for cloning the amplified *cfos* fragment to the Bluescript II vector.

### Immunoblotting

To analyze the expression of IP_3_R1 and TH, various parts of the 8-week-old brain were excised and were sonicated in a Sucrose buffer [0.32 M sucrose, 5 mM Hepes-NaOH (pH = 7.5)] containing the 1x proteinase inhibitors (Roshe). The protein concentrations were measured, and 100 μ g of the samples were lysed with the sample buffer [125 mM Tris-HCl (pH = 6.8), 20% glycerol, 4.0% SDS, 10% 2-mercaptoethanol, 0.1% bromphenol blue], and separated by 7.5% SDS-polyacrylamide gel electrophoresis and transferred to a polyvinyldene difluoride membrane. For c-Fos detection, the striatum of the 4-week-old mice were excised and homogenized with 0.32 M Sucrose buffer, and the nuclear fraction after centrifugation at 2000 rpm was lysed with sample buffer and used for western blotting. Antibodies were rabbit polyclonal anti-TH antibody (1.0 μg/ml, Chemicon), mouse monoclonal anti-β-actin antibody (1.0 μg/ml Sigma), rat monoclonal anti-IP_3_R1 antibody (18A10: 1.0 μg/ml), and rabbit anti-cFos antibody (1.0 μg/ml, Santa cruz).

### Cytochrome oxidase (co) staining

Frozen 4%PFA-fixed 8-week-old brain sections (100 μm thick) were incubated in 0.1 M phosphate buffer containing 4 g sucrose, 50 mg of cytochrome C, and 50 mg of diaminobenzidine per 100 ml of buffer at 37°C for 1–2 h. To compare the CO staining intensities among different genotypes of mice, brains were fixed, cut, and reacted with the same solutions, and the digital photographs were taken on a same day.

### Administration of drugs into the mouse brain (cerebellum, inferior olive, and basal ganglia)

Mice (~2 month-old) were anesthetized with 1.5% halothane anesthesia with N_2_O:O_2_ (3:2) ventilation. A guide cannula (C313, inner diameter: 0.39 mm, outer diameter: 0.71 mm, Plastics One) was implanted at the middle of vermis of cerebellum (1.1 mm in depth). After 3 days of recovery, an internal cannula (1.0 mm projection length from the guide cannula) was replaced with the dummy cannula and PBS or CNQX (5 mM in PBS, Tocris) were infused into the cerebellum at the speed of 0.5 μ l/min for 20 min. The same guide cannula system was used for lidocaine (4.0% in PBS, MP Biomedicals) injection into the IO (the tip of the cannula was targeted to just above the medial nuclei) and bilateral BG (entopeduncular nucleus, 1.3 mm posterior to the Bregma, lateral to 2.2, 4.5 mm depth).

### Electrophysiological recordings using acute cerebellar slice

Cerebellar slices were prepared from *Itpr1*^+/+^ and *Itpr1*^−/−^ mice (P17-20). Parasagittal slices (230 μm thick) of the cerebellar vermis were cut using a vibrating microtome (VT1000S, Leica, Nussloch, Germany) in an ice-cold extracellular solution containing (in mM) 252 sucrose, 3.35 KCl, 21 NaHCO_3_, 0.6 NaH_2_PO_4_, 9.9 glucose, 1 CaCl_2_, and 3 MgCl_2_ and gassed with a mixture of 95% O_2_ and 5% CO_2_ (pH 7.4). The slices were maintained at RT for at least 1 hr in a holding chamber, where they were submerged in artificial cerebrospinal fluid (ACSF) containing (in mM) 138.6 NaCl, 3.35 KCl, 21 NaHCO_3_, 0.6 NaH_2_PO_4_, 9.9 glucose, 2 CaCl_2_, and 1 MgCl_2_ (bubbled with 95% O_2_ and 5% CO_2_ to maintain the pH at 7.4.

PCs were visually identified under Nomarski optics using a water immersion microscope (BX51WI, Olympus, Japan). For loose cell-attached recording, the pipette was gently placed in contact with a cell body of PC, and slight suction was applied. The pipette (2–4 MΩ) containing ACSF was maintained at 0 mV. The membrane currents were recorded using an amplifier, MultiClamp 700B (Molecular Devices, Foster City, CA, USA) and pCLAMP9.2 software (Molecular Devices), digitized, and stored on a computer disk for off-line analysis. All signals were filtered at 2 kHz and sampled at 5–10 kHz. All experiments were performed at 31–32°C. Action potential frequencies were analyzed using the Mini analysis program, version 6 (Synaptosoft, Decatur, GA, USA) and Kyplot 5.0 (Kyence, Tokyo, Japan).

### Extracellular recording in anesthetized mice

Recordings were performed in anesthetized mice (1–2 months old) after an intraperitoneal injection of 50 mg/kg Nembutal using 1.5% halothane anesthesia with N_2_O:O_2_ (3:2) ventilation. Additional doses of 0.15–0.25 mg were given if necessary to maintain anesthetic level. A sedative, chlorprothixene (0.2 mg, i.m.), was administered to supplement of the Nembutal. Atropine (0.3 mg, s.c.) and dexamethasone (0.05 mg, s.c.) were injected subcutaneously (Gordon and Stryker, 1996). The animal's temperature was maintained at 38°C. The heart rate was monitored continuously.

A small hole (1.0 mm) was drilled in the occipital bone above the cerebellar vermis of lobule IV (midline, 4.5 mm caudal from lambda), and the dura was exposed and covered with warm agarose (2.8% in saline). The microelectrode tip (epoxy-coated tungsten microelectrodes, 9–12 MΩ impedance; FHC, ME) was positioned above the small hole and advanced into the cerebellar lobule IV vermis using a stepping motor controlled micromanipulator. Raw signals from the electrodes were amplified, filtered (0.3–5 kHz), digitized at 25 kHz and stored (LabVIEW, National Instrument, Austin, TX). Single and multiunit PC activities (peak heights above the 6 sigma noise level) were isolated by off-line spike sorting (Offline sorter, Plexon, Dallas, TX). Simple spikes (SSs) and complex spikes (CSs) were identified based on their characteristic waveforms.

### EEG, EMG recording

A stainless screw electrode for EEG recording was secured over the cerebellar cortex (2.0 mm posterior to the lambda, on the midline) and a reference screw electrode was placed over the somatosensory (1.5 mm lateral to the midline, 1.0 mm posterior to the bregma) or frontal cortex (1.0 mm lateral to the midline, 2.0 mm anterior to the bregma) (Miyamoto et al., 2012). A stainless wire was inserted in the neck muscle for EMG recording. Polygraphic signals (band-pass filtered at 0.7–170 Hz) were amplified by telemetry system (Data Sciences International, St. Paul, MN) and sampled at 500 Hz (SleepSign, KISSEI COMTEC, Japan). Based on polygraph and infra-red camera monitoring, sleep/waking behavioral state and epileptic EEG pattern was explored.

### Extracellular recording from behaving mice

The tetrodes of four nichrome wires (13 μm) were stereotaxically implanted into the cerebellar vermis of the lobule IV (6.25 mm posterior to the Bregma, on the midline). Signals from each electrode were band-pass filtered (1–6 kHz) and digitized at 25 kHz sampling frequency (Plexon, Dallas, TX). A reference electrode was chosen from electrodes which did not show neuronal activity. Neuronal spike data (firing rate, autocorrelogram, interspike interval) was analyzed by NeuroExplorer (Nex Technologies, Littleton, MA).

Similar to the recording of cerebellar PCs, tetrodes were implanted to monitor neuronal activity in the BG (caudate putamen and globus pallidus, 0.5 mm posterior to the Bregma, 2.0–3.0 mm lateral to the midline, 2.0–4.0 mm depth from the surface) during dystonic movements. Multi-unit neuronal activity data were sampled with a minimal interval of 200 μm by slowly advancing the tetrodes. For behavior analyses, we defined rigid posture as the duration in which mice hunched their backs and extended their paws to maintain the posture. In addition, we defined opisthotonus as an abnormal posture in which the mouse's neck was completely held at the bridging position (i.e., bent fully toward the upper back). We judged the beginning of opisthotonus when the neck was held at the maximal bridging position, and defined the ending as its complete return to the normal (horizontal/unbent) position.

#### Footprint analysis

The hindpaws of 8-week-old *Emx1-Cre;Itpr1^flox/flox^* and *Gpr88-Cre;Itpr1^flox/flox^* mice, or of 19-day-old mice for *Lurcher* experiments, were dipped in non-toxic water-based black paint, and allowed to walk down an enclosed runway lined with white paper, to determine their gait characteristics.

### Statistical analyses

The significance of differences between groups was analyzed using Student's *t*-test, paired Student's *t*-test, Mann-Whitney *U*-test, Dunnett's test, or ANOVA followed by the Bonferroni's test as appropriate. A value of *P* < 0.05 is reported as significant.

## Results

### Enhanced PC activity in IP_3_R1 deficient mice

To examine the neural activities and circuits causing the dyskinetic movements of *Itrp1*^−/−^ mice in detail, we generated several brain-specific IP_3_R1 conditional knockout mice: restricted to the dorsal telencephalon (*Emx1-Cre;Itpr1^flox/flox^*), the cerebellum/brainstem (*Wnt1-Cre;Itpr1^flox/flox^* mice), and to the BG (*Gpr88-Cre;Itpr1^flox/flox^*). Neither *Emx1-Cre;Itpr1^flox/flox^* mice, lacking IP_3_R1 in excitatory neurons and glial cells of the cerebral cortex and hippocampus, nor *Gpr88-Cre;Itpr1^flox/flox^* mice, lacking IP_3_R1 in striatal neurons, exhibited apparent dyskinesia like total *Itpr1*^−/−^ mice (Figure 1B). The *Emx1-Cre;Itpr1^flox/flox^* and *Gpr88-Cre;Itpr1^flox/flox^* mice were born normally and showed normal growth patterns through adulthood. In striking contrast, *Wnt1-Cre;Itpr1^flox/flox^* mice began to show ataxia around postnatal day 9 (P9), and exhibited dyskinesia including opisthotonus, repetitive rigid posture, and tonic contractions of the neck and trunk as they grew beyond 2 weeks (Movie S1, and the footprint analyses shown in Figure 6B, left panel).

**Figure 2:**
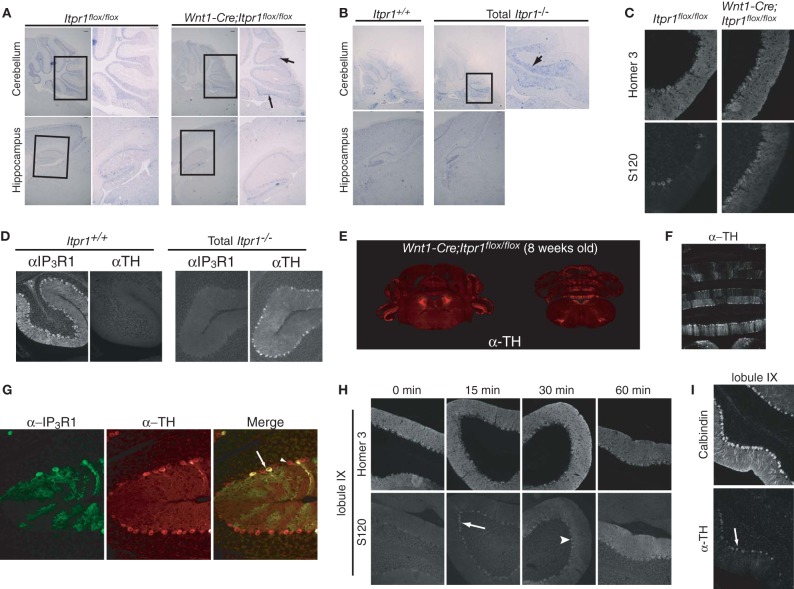
**Abnormal PC activities in the cerebellum of both *Itpr1*^−/−^ and *Wnt1-Cre;Itpr1^flox/flox^* mice**. **(A** and **B**) The *cfos* mRNA expression in the PCs and the hippocampus of 8-week-old *Wnt1-Cre;Itpr1^flox/flox^* **(A)** and 19-day-old *Itpr1*^−/−^ mice **(B)**. Right panel shows magnified inset. Arrow indicates *cfos*-positive soma of PCs. **(C)** Increased phosphorylation level of Homer 3 (S120) in the soma and proximal dendrites of PCs of 8-week-old *Wnt1-Cre;Itpr1^flox/flox^* mice. **(D)** Ectopic expression of tyrosine hydroxylase (TH) in PCs of 19-day-old *Itpr1*^−/−^ mice. **(E)** The TH expression in coronal sections of the *Wnt1-Cre;Itpr1^flox/flox^* cerebellum at 8 weeks. **(F)** Banded patterns of TH expressing PC in the *Wnt1-Cre;Itpr1^flox/flox^* cerebellum at 8 weeks. **(G)** TH expression was not dependent on the IP_3_R1 expression of PCs in *Wnt1-Cre;Itpr1^flox/flox^* mice. **(H)** Intrapenitoneal injection of harmaline (30 mg/kg) increased the signals for the Homer 3 phosphorylation of PCs of 8-week-old wild-type mice in a time dependent manner. **(I)** Intrapenitoneal injection of harmaline increased the TH expression of PCs of wild-type mice after 24 h. All experiments were performed at least three times, and the representative data were shown.

Unlike the premature death in *Itpr1*^−/−^ mice, *Wnt1-Cre;Itpr1^flox/flox^* mice grew to adulthood by hand-feeding. Body weight of *Wnt1-Cre;Itpr1^flox/flox^* mice was about 45% of *Itpr1^flox/flox^* mice at 5 weeks. Cerebellar size of *Wnt1-Cre;Itpr1^flox/flox^* mice at 8 weeks was significantly smaller than that of *Itpr1^flox/flox^* mice, whereas cerebral cortex size was comparable (Figures 1C,D). The apparent morphological constituents of the cerebellum, such as granular layer, PC layer, and molecular layer seemed normal and no apparent cell death occurred as judged by DAPI staining for nuclear condensation. The expression level of IP_3_R1 in the cerebellum of 8 week-old *Wnt1-Cre;Itpr1^flox/flox^* mice was significantly lower than that of *Itpr1^flox/flox^* mice, whereas expression in the hippocampus, striatum, and cerebral cortex of *Wnt1-Cre;Itpr1^flox/flox^* mice was equivalent to that of *Itpr1^flox/flox^* mice (Figures 1E–G). Residual IP_3_R1 expression in the cerebellum of the 8-week-old *Wnt1-Cre;Itpr1^flox/flox^* mice was attributed at least partly to PCs still expressing IP_3_R1 protein after incomplete Cre/flox recombination in the PCs of the *Wnt1-Cre* Tg mice (Figures 1F, 2G).

To further delineate the neurons responsible for the expression of dystonia in *Itpr1*^−/−^ and *Wnt1-Cre;Itpr1^flox/flox^* mice, we investigated the expression of *cfos* mRNA, a neural activity marker (Morgan et al., 1987). Interestingly, we found strong *c-fos* mRNA expression in PCs localized to the caudal parts of the cerebellum in both *Itpr1*^−/−^ and *Wnt1-Cre;Itpr1^flox/flox^* mice, but not in that of *Itpr1*^+/+^ or *Itpr1^flox/flox^* mice (Figures 2A,B, upper panels). No apparent elevation of *cfos* mRNA was observed in the hippocampus and cortex in either *Itpr1*^−/−^ or *Wnt1-Cre;Itpr1^flox/flox^* mice (Figures 2A,B, lower panels). We also examined CaM kinase II-mediated phosphorylation levels of Homer 3 as a marker of PC depolarization (Mizutani et al., 2008). Only weak Homer 3 phosphorylation (S120) signals were observed in the soma and proximal dendrites of PCs in 8-week-old *Itpr1^flox/flox^* mice (Figure 2C), as reported previously (Mizutani et al., 2008). In 8-week-old *Wnt1-Cre;Itpr1^flox/flox^* mice, however, intense Homer 3 phosphorylation was observed at the soma and proximal dendrites, including the apical dendrites of PCs in caudal lobules 9 and 10 (Figure 2C, right).

In addition, we found aberrant tyrosine hydroxylase (TH) expression which is induced by cFos (Nagamoto-Combs et al., 1997) in the *Itpr1*^−/−^ (Figure 2D) and *Wnt1-Cre;Itpr1^flox/flox^* PCs (Figure 2E). The TH-positive PCs were mainly observed in the vermis and flocculus of the *Wnt1-Cre;Itpr1^flox/flox^* and *Itpr1*^−/−^ cerebellum (Figure 2E) and were localized in a banded pattern (Figure 2F). In the wild-type cerebellum, we detected some TH-positive PCs as reported previously (Hess and Wilson, 1991). However, TH expression levels were relatively weak, and the regions expressing TH in a banded manner were both fewer and smaller in size in the *Itpr1*^+/+^ and *Itpr1^flox/flox^* mice than in the *Itpr1* mutant mice (Figure S1). Although PCs normally express a large amount of IP_3_R1 in the brain, the abnormal TH expression in the *Wnt1-Cre;Itpr1^flox/flox^* cerebellum was observed in PCs regardless of IP_3_R1 expression (Figure 2G), suggesting that altered neural inputs onto PCs may trigger the aberrant TH expression.

Since climbing fibers (CF) innervate PCs localized within banded patterns (Oscarsson, 1979), olivocerebellar inputs may be the cause of abnormal involuntary movements in the *Wnt1-Cre;Itpr1^flox/flox^* mice. Harmaline evokes synchronous firing across large populations of PCs via the olivocellebelar pathway by electrical coupling of IO neurons, resulting in the expression of tremor in mice (Llinas and Sasaki, 1989). We intraperitoneally injected harmaline into wild-type mice and examined Homer 3 phosphorylation and TH expression in PCs. We found that IO activation rapidly increased the phosphorylation levels of Homer 3 in the soma and dendrites of PCs in a time dependent manner (Figure 2H). Intense phosphorylation signals were first observed in the soma of PCs within 15 min after injection (Figure 2H, arrow), then progressed into proximal and apical dendrites as time passed (Figure 2H, arrowhead). Furthermore, we observed elevation of TH signals in PCs at the caudal region of the cerebellum, especially lobules XI and X at 24 h after injection (Figure 2I). These results closely resembled those observed in the *Wnt1-Cre;Itpr1^flox/flox^* mice. Therefore, we hypothesized that abnormal PC firing caused by excessive olivocerebellar input is the cause of involuntary movements in the *Itpr1*^−/−^ and *Wnt1-Cre;Itpr1^flox/flox^* mice.

### PC activity correlates with dystonic movements of mice

To reveal the nature of abnormal PC firing underlying the expression of dystonia in the *Itpr1*^−/−^ mice, we first measured spontaneous PC activities by loose cell-attached recording using acute cerebellar slices, in which neuronal inputs from climbing and mossy fibers were severed. Because the PCs highly expressing TH were mainly observed in caudal parts of the cerebellar vermis of *Itpr1*^−/−^ mice (Figure 2E), we measured spontaneous activities of PCs mainly from those areas. However, we found no apparent difference in spike frequency and coefficient of variation (CV) between PCs from wild-type and total *Itpr1*^−/−^ mice under these conditions (Frequency: *Itpr1*^+/+^: 25.89 ± 2.89 (Means ± sem), *n* = 22 cells from 3 mice; *Itpr1*^−/−^: 28.86 ± 3.41, *n* = 26 from 3 mice, Student's *t*-test *P* = 0.51. CV: *Itpr1*^+/+^: 0.23 ± 0.03, *n* = 22; *Itpr1*^−/−^: 0.22 ± 0.02, *n* = 26, Student's *t*-test *P* = 0.72).

**Figure 3:**
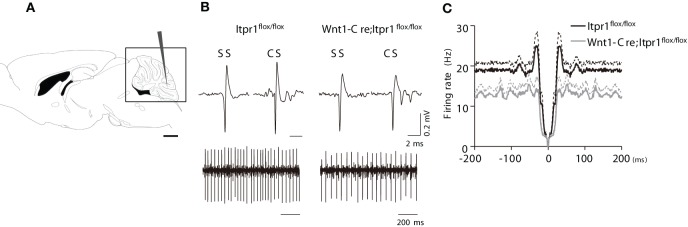
**PC firing in anesthetized *Wnt1-Cre;Itpr1^flox/flox^* mice**. **(A)** Diagram of *in vivo* recording from PCs of the cerebellum. **(B)** Upper panels: representative SSs and CSs of PCs in the *Itpr1^flox/flox^* (left) and *Wnt1-Cre;Itpr1^flox/flox^* (right) mice under anesthesia. Lower panels: recordings of PC spike trains from anesthetized mice. **(C)** Averaged auto-correlograms of PC spikes in the *Wnt1-Cre;Itpr1^flox/flox^* (gray) and *Itpr1^flox/flox^* (black) mice under anesthesia.

Since the cerebellar slice is devoid of neuronal inputs arising from other brain structures, we next asked whether spontaneous PC activities are altered in anesthetized mice. We performed extracellular recording of PC activities from caudal lobules in the cerebellar vermis (Figure 3A), which is responsible for the coordination of body trunk movement. We observed high amplitude PC spiking in both total *Itpr1*^−/−^ and *Wnt1-Cre;Itpr1^flox/flox^* mice as well as in wild-type mice (Figure 3B). Typical SSs and CSs were seen in both total *Itpr1*^−/−^ and *Wnt1-Cre;Itpr1^flox/flox^* mice and were used as an indication of PC activity *in vivo* (Figure 3B). Spontaneous firing rates of *Wnt1-Cre;Itpr1^flox/flox^* mice were decreased compared to *Itpr1^flox/flox^* mice (*Itpr1^flox/flox^*: 17.12 ± 1.61 (*N* = 5 mice, *n* = 37 cells); *Wnt1-Cre;Itpr1^flox/flox^*: 12.84 ± 1.59 (*N* = 4, *n* = 55). Mann-Whitney *U*-test: ^*^P = 0.015.), and this tendency was also observed in total *Itpr1*^−/−^ mice, although this was not significant [*Itpr1*^+/+^: 26.01 ± 3.42 (*N* = 3, *n* = 28); *Itpr1*^−/−^: 19.95 ± 2.52 (*N* = 4, *n* = 25), Student's *t*-test *P* = 0.169]. The CVs were not significantly different between the groups [*Itpr1^flox/flox^*: 1.43 + 0.18 (*n* = 37), *Wnt1-Cre;Itpr1^flox/flox^*: 1.07 + 0.08 (*n* = 55), Mann-Whitney *U*-test: *P* = 0.083, *Itpr1*^+/+^: 1.30 + 0.07 (*n* = 28), *Itpr1*^−/−^: 1.57 + 0.26 (*n* = 25), Mann-Whitney *U*-test: *P* = 0.91].

We also found that averaged auto-correlograms of PC activity in *Wnt1-Cre;Itpr1^flox/flox^* mice lacked a peak around 0-100 ms compared to *Itpr1^flox/flox^* mice (Figure 3C). The peak height (25-35 ms) was significantly lower than that of *Itpr1^flox/flox^* (*Itpr1^flox/flox^*: *n* = 37 from 5 animals; *Wnt1-Cre;Itpr1^flox/flox^* mice: *n* = 55 from 4 animals, Student's *t*-test *P* < 0.01, Figure 3C), suggesting an alteration of PC activity patterns caused by IP_3_R1 deletion. Given that the spontaneous activity pattern was little affected in isolated cerebellar slices, these results suggest that neural inputs to PCs, such as parallel fiber or CF inputs, were changed by IP_3_R1 deletion *in vivo*.

**Figure 4:**
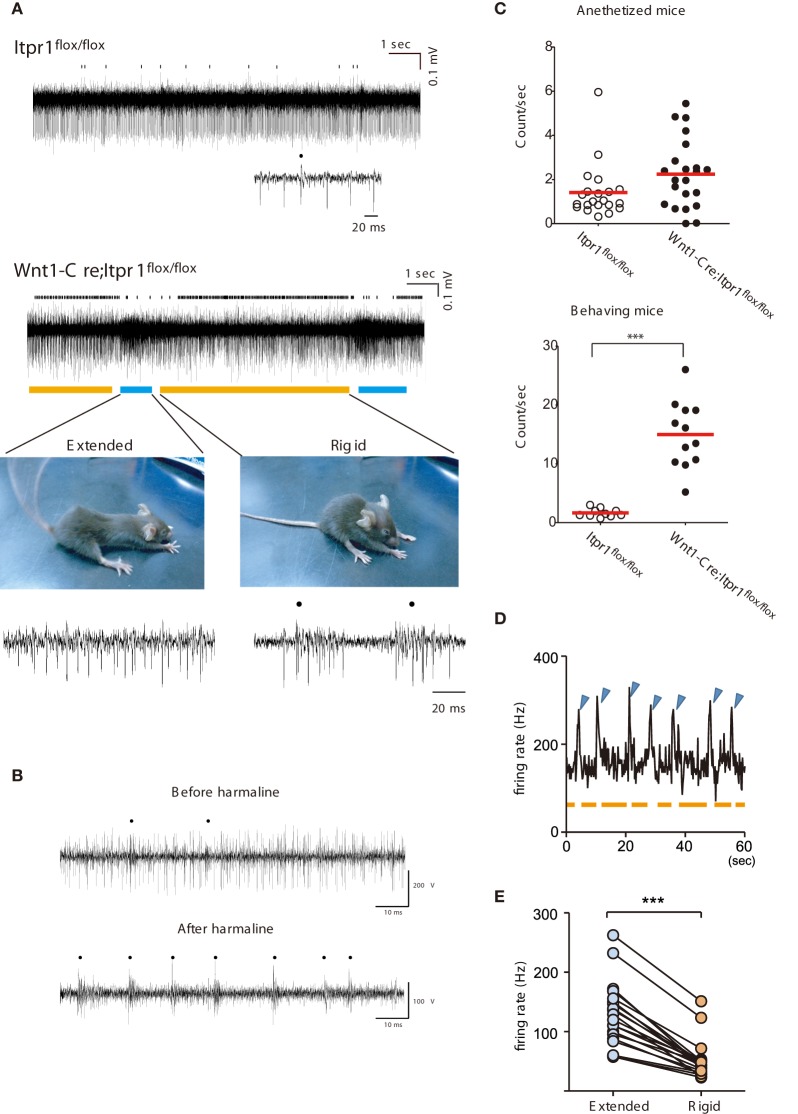
**Correlation of abnormal PC firing with the expression of Dystonia in freely moving *Wnt1-Cre;Itpr1^flox/flox^* mice**. **(A)** Representative recording of PC spiking in freely moving *Itpr1^flox/flox^* and *Wnt1-Cre;Itpr1^flox/flox^* mice. Distinct involuntary movements of *Wnt1-Cre;Itpr1^flox/flox^* mice were highly correlated with change in multi-unit activities of PCs. Bottom picture, representative dyskinetic postures during underlined PC firing periods. Blue line: extension; orange line: compression with rigidity. Dots represent CSs. **(B)** PC spike patterns in wild-type mice before (upper panel) and after (lower panel) intraperitoneal harmaline injection. Dots represent CSs. **(C)** Frequency of CSs in anesthetized (upper panel) and behaving (lower panel) *Itpr1^flox/flox^* and *Wnt1-Cre;Itpr1^flox/flox^* mice. Anesthetized mice, *Itpr1^flox/flox^*: 1.41 ± 0.27 count/s (Mean ± sem. 21 recording sites from 5 animals); *Wnt1-Cre;Itpr1^flox/flox^*: 2.24 ± 0.33 count/s (22 recording sites from 4 animals), *P* = 0.053, Mann-Whitney *U*-test. Behaving mice, *Itpr1^flox/flox^*: 1.61 ± 0.23 (10 recording sites from 4 animals); *Wnt1-Cre;Itpr1^flox/flox^*: 14.96 ± 1.64 (12 recordings form 4 animals), *P* < 0.0001, Mann-Whitney *U*-test. **(D)** Relationship between firing rate and two postures. Blue arrowhead: extension; orange: shrinkage with rigidity. **(E)** Population firing data. (*N* = 4 mice, *n* = 20 recording sites, paired Student's *t*-test ^***^P < 0. 0001).

Anesthetics influence synaptic neurotransmission or cellular communication (Keane and Biziere, 1987) and suppress animal behavior and movement. To gain more insight into the possible link between PC firing and the expression of dystonia, we recorded multiple unit activity of PCs from freely moving mice (Figure 4A). High amplitude putative PC spiking and low amplitude background activity were alternately recorded as the electrode advanced (400–800 μm). Multiple spiking and increased background activity of awake animals sometimes made discrimination between SSs and CSs difficult. In *Itpr1^flox/flox^* mice, putative PC activity showed regular tonic firing similar to the firing pattern of anesthetized mice (Figure 4A, upper panel). We did not see a drastic change of firing rate associated with particular movements or behaviors in the caudal part of the vermis. Likewise, sleep-wake state associated changes of PC firing rate were not evident.

In contrast, a sharp increase of firing rates was observed intermittently (about once/10 s) in *Wnt1-Cre;Itpr1^flox/flox^* mice and was tightly coupled to body movement related to paroxysmal dyskinesia (Figure 4A). Typically, *Wnt1-Cre;Itpr1^flox/flox^* mice gradually increased rigidity during the low firing period (indicated by the orange bar in Figure 4A, lower right panel). Then, they abruptly extended their trunk and limbs simultaneously (Figure 4A, lower left panel) during the high frequency period (indicated by the blue bar). This sequence of high and low frequency firing recurred while the animal was awake, but not during stiff ambulation. Though clear isolation of CSs was difficult, CS activity prevailed during the rigid posture (the orange bar), while SS activity became dominant with high frequency firing during the body-extension (the blue bar) (Figure 4A, lower panels).

Interestingly, the intraperitoneal injection of harmaline (30 mg/kg) in awake wild-type mice reduced SS, and caused CS-dominant spike patterns in PCs (Figure 4B) reminiscent of their firing patterns in the *Wnt1-Cre;Itpr1^flox/flox^* mice during rigid posture. When *Wnt1-Cre;Itpr1^flox/flox^* mice fell asleep, these particular patterns of PC activity diminished. The frequency of CSs was significantly increased in behaving *Wnt1-Cre;Itpr1^flox/flox^* mice as compared to *Itpr1^flox/flox^* mice, whereas the difference was not evident under anesthetized condition (Figure 4C). An increase of multiunit activity corresponded to the body-extension phase (high firing rate period, blue arrowhead) as judged by an independent observer (Figure 4D). Multiunit firing peaks in several recordings from lobules in caudal portions of the vermis were averaged (*N* = 4, 20 MUA recordings), and we confirmed distinct cerebellar activity changes associated with rigid and extended postures (Figure 4E).

### Temporal inactivation of the cerebellum ameliorates dystonia

**Figure 5:**
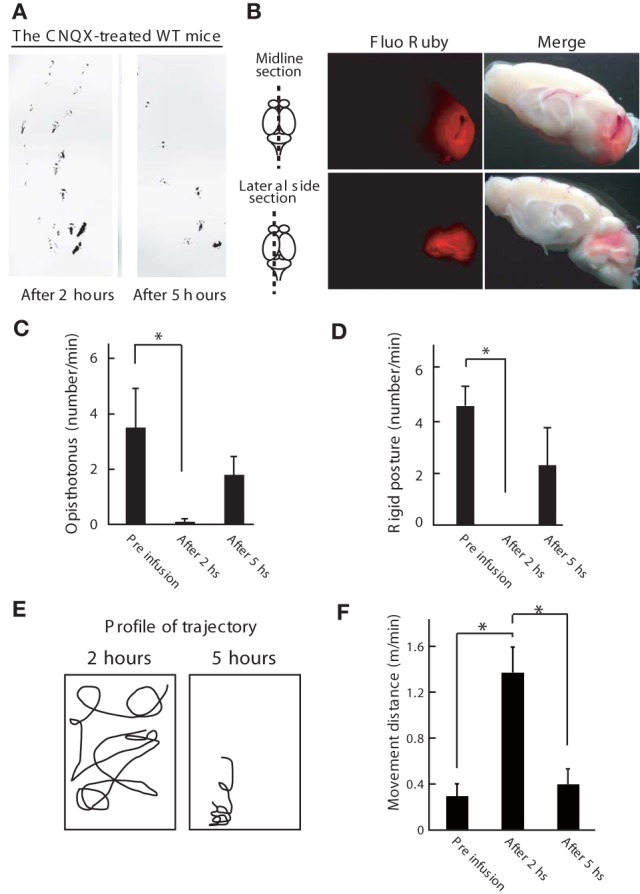
**Inhibition of cerebellum activity ameliorates the dystonic movements of *Wnt1-Cre;Itpr1^flox/flox^ mice***. **(A)** Footprint of wild-type mice at 2 and 5 h after the CNQX infusion into the cerebellum. The mice hind paws were visualized with non-toxic ink. Note that gait abnormality at 2 h later after infusion. **(B)** Gross appearance of the brain infused with Fluo Ruby after 2 h of infusion. Strong Fluo Ruby signals at cerebellum were detected in the midline and lateral side sections. **(C–F)** Cerebellar AMPA receptor blockade (CNQX infusion) improved voluntary movement of *Wnt1-Cre;Itpr1^flox/flox^* mice. Incidence of abnormal postures **(C**: Opistothonus, **D**: Rigid posture with freezing as shown in Figure 4A) were scored. Means ± sem. Opistothonus, pre-infusion: 3.57 ± 1.21; after 2 h: 0.10 ± 0.10, after 5 h: 1.80 ± 0.64, Dunnett's test ^*^P < 0.05; Rigid posture, pre-infusion: 4.50 ± 0.76; after 2 h: 0, after 5 h: 2.23 ± 1.50, Dunnett's test ^*^P < 0.05 (*N* = 3). **(E)** Representative trajectory over 2 min. Box represents 34 × 38 cm square. **(F)** locomotor distance. Means ± sem. Pre infusion: 0.30 ± 0.11; after 2 h: 1.37 ± 0.01; after 5 h: 0.40 ± 0.016. ^*^P < 0.05. ANOVA, followed by Bonferroni's test. (*N* = 3).

To confirm the involvement of cerebellar activity in the dystonic movements of *Wnt1-Cre;Itpr1^flox/flox^* mice, we inhibited cerebellar activity by α-amino-3-hydroxy-5-methyl-4-isoxazolepropionic acid (AMPA) receptor antagonist (CNQX) infusion. Inactivation of the cerebellum was confirmed by ataxia of CNQX-infused wild-type mice (Figure 5A), including abnormal footprints with shorter step length and wider gait after 2 h of infusion that recovered by 5 h (Figure 5A). Cerebellum specific infusion of the drug was confirmed by Fluo Ruby (Figure 5B). No ataxic gait was observed in wild-type mice infused with saline. Strikingly, CNQX infusion into the cerebellum of *Wnt1-Cre;Itpr1^flox/flox^* mice improved their voluntary movement significantly: dyskinesia such as opisthotonus, rigid posture, and tremor was abolished (Figures 5C,D). Although the mutant mice still showed ataxia, they exhibited partially restored gait within 2 h (Figures 5E,F; Movie S2). After 5 h, dystonic movements of *Wnt1-Cre;Itpr1^flox/flox^* mice appeared.

### Genetic deletion of PCs rescues dystonia of *Wnt1-Cre;Itpr1*^flox/flox^ mice

**Figure 6:**
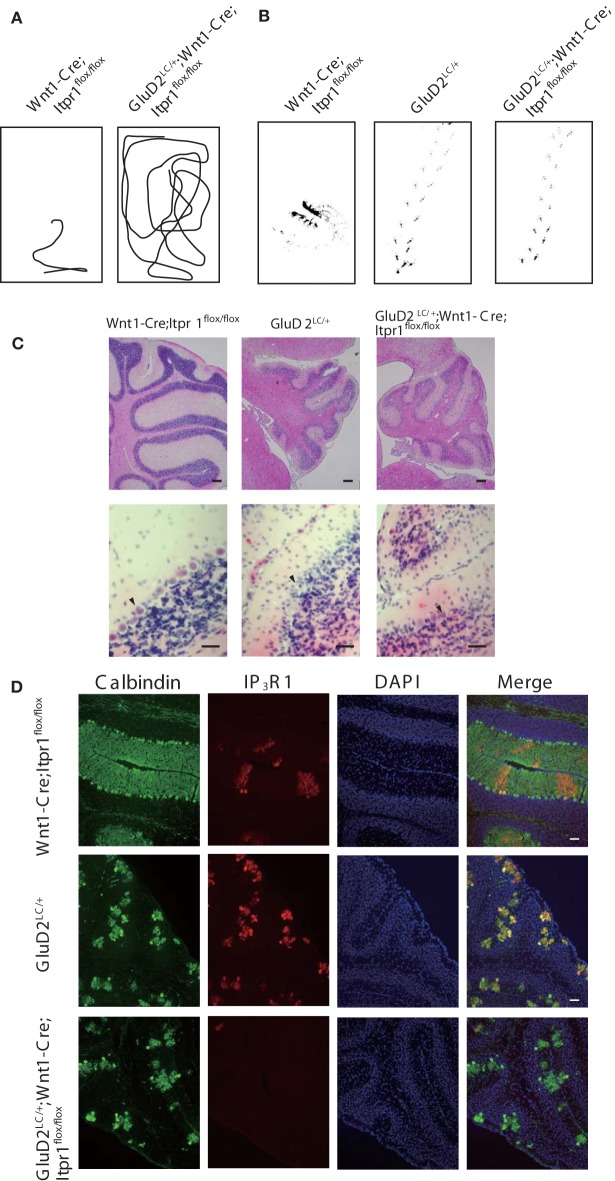
**Genetic deletion of PC rescues dystonia of *Wnt1-Cre;Itpr1^flox/flox^* mice**. **(A)** Representative trajectories over 2 min of *Wnt1-Cre;Itpr1^flox/flox^* and *GluD2^LC/+^;Wnt1-Cre;Itpr1^flox/flox^* mice at postnatal 19 days after birth. Box represents 17 × 26 cm square. **(B)** Representative footprints of *Wnt1-Cre;Itpr1^flox/flox^*, *GluD2^LC/+^*, and *GluD2^LC/+^;Wnt1-Cre;Itpr1^flox/flox^* mice at 19 days old. Animal's hind paw prints were visualized with non-toxic ink. **(C)** Morphological assessment of cerebellar PC deletion by HE staining at 23 days old. **(D)** Immunohistochemistry of the IP_3_R1 and Calbindin expression in the cerebellum from *W*nt1-Cre;Itpr1^flox/flox^, *GluD2^LC/+^*, and *GluD2^LC/+^;Wnt1-Cre;Itpr1^flox/flox^* mice at 23 days old.

To further explore the influence of cerebellar output from PCs on dystonic movements, we also genetically deleted PCs from the cerebellum of *Wnt1-Cre;Itpr1^flox/flox^* mice by mating them with *Lurcher* mice (*GluD2^LC/+^)* in which most of PCs die due to a mutation of the delta 2 glutamate receptor (*GluD2*) during the second postnatal week (Barmack and Yakhnitsa, 2003). Interestingly, we found that dystonic movements in *Wnt1-Cre;Itpr1^flox/flox^* mice were completely abolished in *GluD2^LC/+^;Wnt1-Cre;Itpr1^flox/flox^* mice. The *GluD2^LC/+^;Wnt1-Cre;Itpr1^flox/flox^* mice greatly improved their gait to a level similar to those of *GluD2^LC/+^* mice [Figures 6A,B, and Movie S3, *N* = 4. Stride length: *GluD2^LC/+^*: 1.48 ± 0.053 and *GluD2^LC/+^;Wnt1-Cre;Itpr1^flox/flox^*: 1.60 ± 0.03 (Mean ± sem, Student's *t*-test *P* = 0.06, *n* = 18 from 3 mice), base width: *GluD2^LC/+^*: 1.03 ± 0.014 and *GluD2^LC/+^;Wnt1-Cre;Itpr1^flox/flox^*: 0.83 ± 0.043 (Mean ± sem, Student's *t*-test *P* < 0.001, *n* = 12 from 3 mice)]. Loss of most of PCs were confirmed in the cerebellum of *GluD2^LC/+^;Wnt1-Cre;Itpr1^flox/flox^* mice (Figures 6C,D). These results strongly suggested that abnormal cerebellar output from PCs produces dystonia in mice lacking IP_3_R1.

### Olivo-cerebellar pathway, but not BG, is involved in the expression of dystonia

**Figure 7:**
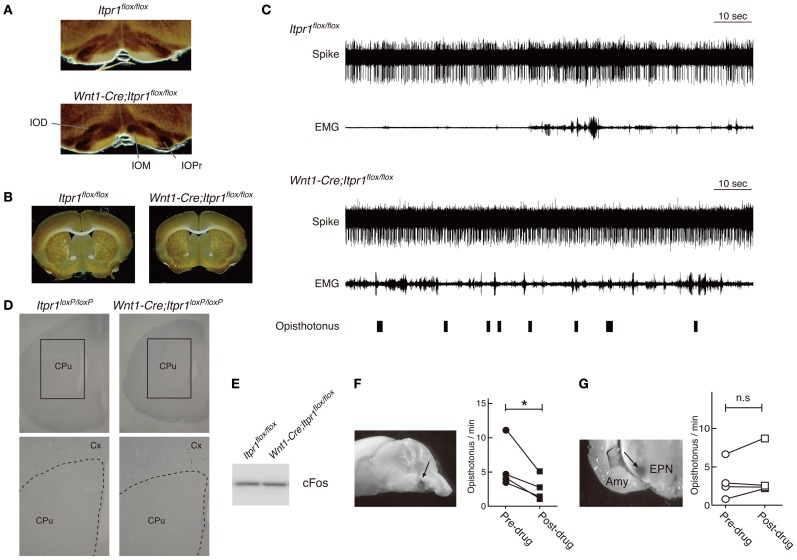
**Inhibition of IO but not BG activity ameliorates the dystonic movements of *Wnt1-Cre;Itpr1^flox/flox^* mice**. **(A** and **B**) CO staining of IO **(A)** and BG **(B)** of 8 week-old *Itpr1^flox/flox^* and *Wnt1-Cre;Itpr1^flox/flox^* mice. **(C)** BG spiking activity and EMG in *Itpr1^flox/flox^* and *Wnt1-Cre;Itpr1^flox/flox^* mice. Single-unit activity (SUA). *Itpr1^flox/flox^*: 8.523 ± 1.669 (*n* = 20). *Wnt1-Cre;Itpr1^flox/flox^*: 15.05 ± 4.398 (*n* = 22). Mean ± sem. Student's *t*-test *P* = 0.19. For *Wnt1-Cre;Itpr1^flox/flox^* mice, opisthotonus was presented by a hand swich. **(D)** The expression level of *cfos* mRNA in the striatum of 19-day-old *Itpr1^flox/flox^* and *Wnt1-Cre;Itpr1^flox/flox^* mice. CPu: caudate putamen. Cx, cerebral cortex. *N* = 3. The representative data was shown. **(E)** The expression level of cFos in the striatum of *Itpr1^flox/flox^* and *Wnt1-Cre;Itpr1^flox/flox^* mice at 4 weeks old. Fifty μ g of proteins were loaded for each lane. The representative data was shown (*n* = 3). **(F)** Effect of lidocaine infusion into IO on the opisthotonus of *Wnt1-Cre;Itpr1^flox/flox^* mice. Left panels show the infusion site visualized with methylene blue. Right panels show the number of opisthotonus before and after lidocaine infusion (*N* = 4). ^*^Paired Student's *t*-test *P* < 0.05. **(G)** Effect of lidocaine infusion into bilateral BG (entopeduncular nucleus) on opisthotonus number of *Wnt1-Cre;Itpr1^flox/flox^* mice (*N* = 4). Amy, amygdala; EPN, entopeduncular nucleus; n.s, not significant determined by paired Student's *t*-test.

Because predominant CS activities prevailed during dystonic posture (Figure 4), we checked activities of IO neurons, which send CFs to PCs, by a cytochrome oxidase (CO) assay. We found that the CO-staining intensities in the IO of *Wnt1-Cre;Itpr1^flox/flox^* mice were increased as compared with those of *Itpr1^flox/flox^* mice (Figure 7A, Relative CO activity. Principal nuclei (IOPr), *Itpr1^flox/flox^*: 0.97 ± 0.03, *Wnt1-Cre;Itpr1^flox/flox^*: 1.17 ± 0.05, *P*< 0.05; medial inferior olive (IOM), *Itpr1^flox/flox^*: 0.68 ± 0.02. *Wnt1-Cre;Itpr1^flox/flox^*: 1.64 ± 0.1, *P* < 0.0001; and dorsal accessory inferior olive (IOD), *Itpr1^flox/flox^*: 0.64 ± 0.02, *Wnt1-Cre;Itpr1^flox/flox^*: 0.95 ± 0.06, Means ± sem, Student's *t*-test *P* < 0.01, *n* = 6 from 3 mice). Inferior olive IP_3_R1 expression was below the threshold of immunohistochemical detection even in wild-type mice, most likely because of its significantly lower expression relative to hippocampal, striatal, and cerebral cortical neurons. In contrast, we did not detect a significant difference in the CO staining intensities of the BG between *Wnt1-Cre;Itpr1^flox/flox^* and *Itpr1^flox/flox^* mice (Relative CO activity. *Itpr1^flox/flox^*: 1.02 ± 0.02; *Wnt1-Cre;Itpr1^flox/flox^*: 0.98 ± 0.03, Student's *t*-test *P* = 0.20, *n* = 6 from 3 mice) (Figure 7B). In addition, contrary to the PC activity patterns, we did not observe distinct correlations between BG spiking activity and dystonic movement and activity patterns were essentially indistinguishable (Figure 7C). Activity levels of neurons in the *Wnt1-Cre;Itpr1^flox/flox^* BG were also similar to those of *Itpr1^flox/flox^* BG (firing rate of the BG neurons, single-unit activity [*Itpr1^flox/flox^*: 8.523 ± 1.669 (*n* = 20). *Wnt1-Cre;Itpr1^flox/flox^*: 15.05 ± 4.398 (*n* = 22). Mean ± sem. Student's *t*-test *P* = 0.19.]. In addition, the expression levels of *cfos* mRNA and cFos in the striatum were comparable between *Itpr1^flox/flox^* and *Wnt1-Cre;Itpr1^flox/flox^* mice (Figures 7D,E).

To examine whether altered IO activity was associated with dystonia in the *Wnt1-Cre;Itpr1^flox/flox^* mice, we pharmacologically inhibited IO activities. We found that lidocaine injection into IO decreased opisthotonus of *Wnt1-Cre;Itpr1^flox/flox^* mice (Figure 7F), although tremor of limbs and ataxia were still observed (Movie S4). In contrast, pharmacological inhibition of bilateral BG (entopeduncular nucleus) activity by lidocaine injection did not significantly affect frequency of opisthotonus in *Wnt1-Cre;Itpr1^flox/flox^* mice (Figure 7G; Movie S5). These results suggested that altered activities of olivocerebellar tracts cause dystonia in *Wnt1-Cre;Itpr1^flox/flox^* mice in a BG-independent manner.

## Discussion

In this study, we demonstrated that genetic deletion of IP_3_R1 within cerebellum and brainstem is sufficient to cause dystonia in mice, and that further pharmacological inactivation of the cerebellum or the IO and deletion of PCs ameliorate the dyskinesia. Thus, our data suggested that dystonia is a gain of function rather than loss of function of olivocerebellar pathways, which is in line with the previous findings (Campbell et al., 1999; Pizoli et al., 2002). Moreover, using electrophysiological recordings of PC activity from freely behaving dystonic mice, we have also demonstrated the relationship between temporal changes of PC spike activity possibly triggered by altered IO activation and the expression of dystonia. Although altered PC activity was found in the movement-restricted dystonic rat (Ledoux and Lorden, 2002), how the temporal changes of PC firing patterns are related to ongoing dystonic movements were unknown. We revealed a distinct pattern of PC firing in freely moving *Wnt1-Cre;Itpr1^flox/flox^* mice during distinct dystonic postures, which could not be observed in neither the anesthetized preparation nor the cerebellar slices. During dystonic movements, PC activities exhibiting repetitive CS patterns were predominant. Since CSs are thought to be important for voluntary movements (Welsh et al., 1995; Kitazawa et al., 1998; Welsh, 2002), the repetitive abnormal synchronized CSs with high frequency during rigid posture may in part underlie dystonia.

Chen et al. recently reported the low-frequency oscillations of flavoprotein autofluorescence in the cerebellar cortex of tottering mice (Chen et al., 2009), and showed that the oscillation was accentuated during dystonia. However, the cellular types and mechanisms that contribute to the enhancement of the oscillation in the mutant mice were unknown. By measuring the PC activities from behaving *Wnt1-Cre;Itpr1^flox/flox^* mice, here we found a precise temporal association between CS-dominant PC firings and distinct dystonic movements. Thus, increase of CF frequency through IO activation may underlie the expression of dystonia in *Wnt1-Cre;Itpr1^flox/flox^* mice. This hypothesis is in line with our finding that infusion of AMPAR blocker in the cerebellum ameliorates dystonia in *Wnt1-Cre;Itpr1^flox/flox^* mice, because AMPA receptor blocker inhibits CF-PC synapse transmission. Although we don't know the relationship between the CS-dominant PC firings in the present study and the low-frequency oscillations in cerebellar cortex shown in the Chen's paper, the CS-dominant PC firings is most likely to be independent of the cerebellar oscillation, since the oscillation was reported to be intrinsic to the cerebellar cortex and the cerebellar blockade by AMPA receptor or by electrical stimulation of PFs did not affect the oscillation (Chen et al., 2009).

Our results also suggest a previously unknown pathogenesis of dystonia induced by abnormal cerebellar activity in mice, namely BG-independent dystonia, based on the following facts; no apparent motor abnormality of BG-specific IP_3_R1 conditional mice, no difference in CO staining intensity in *Itpr1^flox/flox^* and *Wnt1-Cre;Itpr1^flox/flox^* mouse's BG, little correlations of BG activity and dystonic movement, and the ineffectiveness of pharmaceutical BG inactivation on dystonia of *Wnt1-Cre;Itpr1^flox/flox^* mice. Thus, we propose that altered cerebellar activity causes dystonia by a mechanism, which does not involve BG activity in *Wnt1-Cre;Itpr1^flox/flox^* mice. It is possible that the abnormal cerebellar outputs generated by IO might be directly sent to spinal cords via red nucleus or reticular formation. It is also worth mentioning that the distinctive CSs appeared only in awake *Wnt1-Cre;Itpr1^flox/flox^* mice, and that altered activation of IO itself was not sufficient for generation of dystonia, since harmaline, which evokes similar CS dominant spike patterns of PCs, does not cause dystonia. Therefore, uncoordinated timings between voluntary corticospinal signals and the involuntary cerebellar-reticulospinal signals generated by IO activation with spinocerebellar (somatosensory) inputs may cause simultaneous activation of agonist- and antagonist muscles, leading to dystonia in the *Wnt1-Cre;Itpr1^flox/flox^* mice.

In sum, our study suggests that BG-independent dystonia is triggered by abnormal cerebellar outputs in mice. *Wnt1-Cre;Itpr1^flox/flox^* mice may provide a therapeutic dystonia model solely dependent upon abnormal neural activities within the cerebellum and brainstem. Recently, it was reported that a deletion of the *Itpr1* gene is associated with involuntary movements in patients of spinocerebellar ataxia type 15 (Di Gregorio et al., 2010; Marelli et al., 2011), which has been thought to be pure cerebellar ataxia (Hara et al., 2008). The above involuntary movements may be dystonia-related, given that dystonia can be a prominent symptom in SCAs, including some cases with exclusively cerebellar pathology (Manto, 2005). However, since spinocerebellar ataxia type 15 is a slow progressive autosomal dominant disease exhibiting cerebellar atrophy with PC death (Knight et al., 2003; Gardner et al., 2005), severe dystonia would not happen in human. Nevertheless, IP_3_R is known to interact with Na-K ATPase, a causal gene for DYT12 dystonia, and a Na-K ATPase inhibitor, ouabain, causes aberrant Ca^2+^ release from the IP_3_Rs (Zhang et al., 2006). Thus, it is possible that dysfunction of IP_3_R1 could be associated with dystonia in human. Further studies on the mechanism by which disturbed Ca^2+^ signals from IP_3_R1 lead to the repetitive synchronized CSs in our mutant mice, such as potential Ca^2+^-dependent regulation of gap junction among IO neurons, may contribute to the understanding of pathogenesis and the development of new therapies for dystonia.

## Author contributions

Chihiro Hisatsune designed the project, performed experiments, and wrote the manuscript. Katsuhiko Mikoshiba wrote the manuscript. Hiroyuki Miyamoto, Moritoshi Hirono, Takao K. Hensch, and Masahisa Yamada. performed the electrophysiological experiments and wrote the manuscript. Naoko Ogawa, Etsuko Ebisui, and Takeyuki Sugawara performed the experiments. Naohide Yamaguchi, and Mitsuharu Hattori generated the *Itpr1^flox/+^* mice. Toshio Ohshima helped to establish mutant mice.

## Conflict of interest statement

The authors declare that the research was conducted in the absence of any commercial or financial relationships that could be construed as a potential conflict of interest.

## Supplementary material

Movie S1 **Behavior of *Wnt1-Cre;Itpr1^flox/flox^* mice**.

Movie S2 **Behavior of *Wnt1-Cre;Itpr1^flox/flox^* mice before and 2 h after CNQX infusion**.

Movie S3 **Behavior of *GluD2^LC/+^;Wnt1-Cre; Itpr1^flox/flox^* mice**.

Movie S4 **Behavior of *Wnt1-Cre;Itpr1^flox/flox^* mice before and after lidocaine injection into the inferior olive**.

Movie S5 **Behavior of *Wnt1-Cre;Itpr1 ^flox/flox^* mice before and after lidocaine injection into the BG**.
