# Supplementary material for: IP3R1 deficiency in the cerebellum/brainstem causes basal ganglia-independent dystonia by triggering tonic Purkinje cell firings in mice
Source: Front Neural Circuits. 2013 Oct 4;7:156. doi: 10.3389/fncir.2013.00156 (PMC3790101; doi:10.3389/fncir.2013.00156)
Supplement: Supplementary file 6 [file 63003_Hisatsune_Presentation1.PDF]

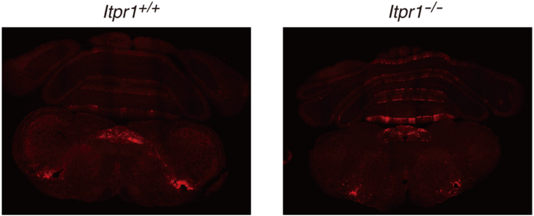

Figure S1. The TH expression in the cerebellum of 20 day-old *Itpr1*$^{+/+}$ and *Itpr1*$^{-/-}$ mice. The coronal section of the cerebellum was stained with anti-TH antibody.
